# A review of psoriasis image analysis based on machine learning

**DOI:** 10.3389/fmed.2024.1414582

**Published:** 2024-08-07

**Authors:** Huihui Li, Guangjie Chen, Li Zhang, Chunlin Xu, Ju Wen

**Affiliations:** ^1^School of Computer Science, Guangdong Polytechnic Normal University, Guangzhou, China; ^2^The Second School of Clinical Medicine, Southern Medical University, Guangzhou, China; ^3^Department of Dermatology, Guangdong Second Provincial General Hospital, Guangzhou, China

**Keywords:** machine learning, deep learning, dermatology, psoriasis, review

## Abstract

Machine Learning (ML), an Artificial Intelligence (AI) technique that includes both Traditional Machine Learning (TML) and Deep Learning (DL), aims to teach machines to automatically learn tasks by inferring patterns from data. It holds significant promise in aiding medical care and has become increasingly important in improving professional processes, particularly in the diagnosis of psoriasis. This paper presents the findings of a systematic literature review focusing on the research and application of ML in psoriasis analysis over the past decade. We summarized 53 publications by searching the Web of Science, PubMed and IEEE Xplore databases and classified them into three categories: (i) lesion localization and segmentation; (ii) lesion recognition; (iii) lesion severity and area scoring. We have presented the most common models and datasets for psoriasis analysis, discussed the key challenges, and explored future trends in ML within this field. Our aim is to suggest directions for subsequent research.

## 1 Introduction

Psoriasis is a chronic, inflammatory and hyperproliferative skin disease with a genetic basis (1). It can appear in any form on the arms, legs, scalp, buttocks, the folds of the skin and the trunk of the body (2). Awareness is increasing that psoriasis as a disease is more than skin deep and that it is associated with systemic disorders, including Crohn's disease, diabetes mellitus (notably type 2), metabolic syndrome, depression, and cancer (3). The disease follows a lengthy course and is prone to relapse, sometimes persisting for a lifetime. Psoriasis is characterized by scaling, silver shavings, protrusion and erythema. Its severity is evaluated based on the degree of infiltration, erythema, area, epidermal desquamation/scaling and other indicators, each of which is scored according to different clinical manifestations (4). Worldwide, approximately 125 million people have psoriasis, and psoriasis prevalence is highly variable across regions, ranging from 0.5% in parts of Asia to as high as 8% in Norway. In most regions, women and men are affected equally (5).

ML has been widely developed to analyse health data, particularly medical images, to assist professionals in making decisions and reducing medical errors. In particular, DL applications have shown promising results in dermatology and other specialties, including radiology, cardiology, and ophthalmology (6). ML technologies can be broadly classified into TML and DL. In TML, data features are obtained through a feature engineering process and then fed into a classifier for result prediction. Common TML classifiers include Random Forest (RF) (7), K-means (8), Decision Tree (9) K-Nearest Neighbor (KNN) (10) and Support Vector Machine (SVM) (11). For instance, a random forest is a decision-making process, whereas KNN classifies vectors with similar distances in a feature space into the same class. Although these techniques are easy to explain and intuitive, they become less effective as the complexity of the data increases.

With the upgrading of algorithms and hardware, researchers began to focus on DL and explore its advantages in medical image analysis (12). DL has significant advantages in dermatological medical image processing: (1) Automatic feature extraction; (2) Handle complex data; (3) High performance. Convolutional neural networks (CNNs) are commonly used in the selection of DL models for dermatological diagnosis. Several CNNs-based models, including U-Net (13) and ResNet (14), have been used for psoriasis analysis. However, despite the strong potential of deep learning in skin medical image processing, it also faces challenges, such as data scarcity leading to model overfitting, complex models leading to long training times, and inexplicable models making it difficult for doctors to trust their results (15). Moreover, for DL, the deeper the layers of the model, the higher the hardware requirements, and the DL spend will be higher compared to TML.

Although recent studies have reviewed the application of AI in psoriasis diagnosis (16–19), these reviews did not conduct a thorough analysis of the ML models and the associated datasets. Therefore, this paper provides a detailed review of the use and advantages and disadvantages of ML models (including TML and DL models) in the application of psoriasis diagnosis. The contributions of this review can be summarized as follows:

- Provides a comprehensive overview of ML models used in psoriasis diagnosis, including TML models and DL models, and provides a detailed analysis of the advantages and disadvantages of each model.
- Evaluates existing psoriasis datasets and discusses their limitations in model development and evaluation.
- Proposes some future research directions to improve the accuracy and efficiency of psoriasis diagnosis.

The rest of this article is organized as follows: Section 2 introduces the methods adopted in this paper to conduct systematic review research; Section 3 introduces the results of paper retrieval. In Section 3.1, we introduce several publicly accessible datasets; The key content of this review, that is, the tasks of machine learning in various psoriasis analyses, are presented in Section 3.2, of which Section 3.2.1 is the segmentation task, Section 3.2.2 is the recognition task, and Section 3.2.3 is the assessment task. Section 4 is the discussion, including the challenges in Section 4.2 and future developments in Section 4.3; Finally, a systematic summary of this paper is given in Section 5.

## 2 Methods

We performed a literature search for relevant publications in 3 databases: Web of Science, PubMed, and IEEE Xplore. We chose these databases in order to cover general resources (Web of Science), medical (PubMed), and computing (IEEE Xplore). Relevant articles published in English between 2014 and April 2024, were considered. We use “and/or” operators to combine multiple keywords with “psoriasis”, including “Machine Learning (ML)”, “Deep Learning (DL)”, “segmentation”, “recognition”, “assessment”, and “review”. To avoid missing keywords, we expanded the search scope of keywords to the entire text. Search expressions are shown in Table 1.

**Table 1:** Search expressions used in the systematic review.

**Database**	**Query statement**	**Year of release**
Web of Science	ALL=(psoriasis) AND (ALL=(ML) OR ALL=(DL))	2014–2024.04
PubMed	ALL=(psoriasis) AND (ALL=(ML) OR ALL=(DL)) AND (ALL=(segmentation) OR ALL=(recognition) OR ALL=(assessment))	
IEEE Xplore	ALL=(skin) AND ALL=(review) AND (ALL=(ML) OR ALL=(DL))	

**Figure 1:**
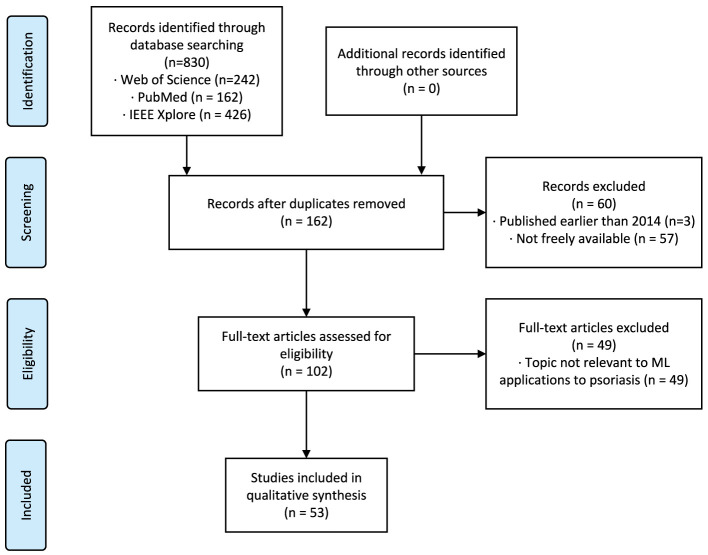
Systematic review flowchart according to the PRISMA framework. PRISMA indicates Preferred Reporting Items for Systematic Reviews and Meta-Analyses.

We reviewed all retrieved papers from all platforms and removed duplicates, non-English papers, papers published before 2014, inaccessible papers, papers not related to machine learning, and papers not related to psoriasis. The remaining papers were confirmed by the authors to meet the requirements and were finally included in the review. Figure 1 reports our systematic review process using the Preferred Reporting Items for Systematic Reviews and Meta-Analyses framework (20).

## 3 Results

**Figure 2:**
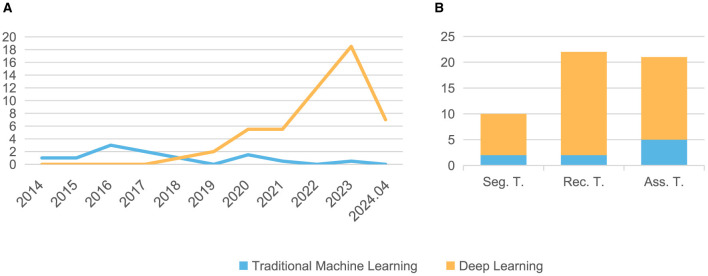
The distribution of the papers summarized in this article. **(A)** Number of papers published each year from 2014 to 2024.04; **(B)** Number of papers related to three different tasks. Seg, Segmentation; Rec, Recognition; Ass, Assessment; T, Task.

Our search method identified 830 citations. After following the review protocal, 53 full-text articles were included for qualitative synthesis (Figure 1). Following the models used in the papers and the year of publication (Figure 2A), we found that the number of studies on psoriasis on machine learning has increased in recent years, a trend that can be attributed to the increase in datasets and advances in modeling. In all, we summarized a total of 10 papers on psoriasis lesion segmentation, 22 papers on psoriasis lesion recognition, and 21 papers on psoriasis severity scoring (Figure 2B). This review provides a comprehensive analysis of these papers and the datasets they use, describing the progress, limitations, and future directions of psoriasis in ML research.

### 3.1 Datasets

To conduct psoriasis analysis using ML, psoriasis data and various labels are necessary. After reviewing a significant amount of psoriasis-related literature, we discovered that most of it is produced in collaboration with hospitals and the datasets are private. As can be seen from the Table 2, from paper to paper they vary in the number of images, the source of the images and even the way the images are captured. This makes it impossible to compare these studies peer-to-peer, but only independently.

**Table 2:** Statistics of private datasets adopted by the reviewed articles.

**References**	**Number of images for various tasks and classes**							
	**Seg. task**	**Rec. task**		**Ass. task**				
	**Images**	**Pso**	**No-Pso**	**H**.	**Mi**.	**Mo**.	**Se**.	**V.Se**.
George et al. (21)	676	-	-	-	-	-	-	-
Dash et al. (22)	5,179	-	-	-	-	-	-	-
Shrivastava et al. (23)	-	270	270	-	-	-	-	-
Zhao et al. (24)	-	900	7,121	-	-	-	-	-
Hammad et al. (25)	-	2,055	1,677	-	-	-	-	-
Shrivastava et al. (26)	-	-	-	383	47	245	145	28
Shrivastava et al. (27)	-	-	-	218	29	138	165	121
Dash et al. (28)	5,000	5,000	5,000	5,000	845	1,404	1,465	1,286

Pso, Psoriasis; H., Health; Mi., Mild; Mo., Moderate; Se., Severe; V.Se., Very Severe.

**Figure 3:**
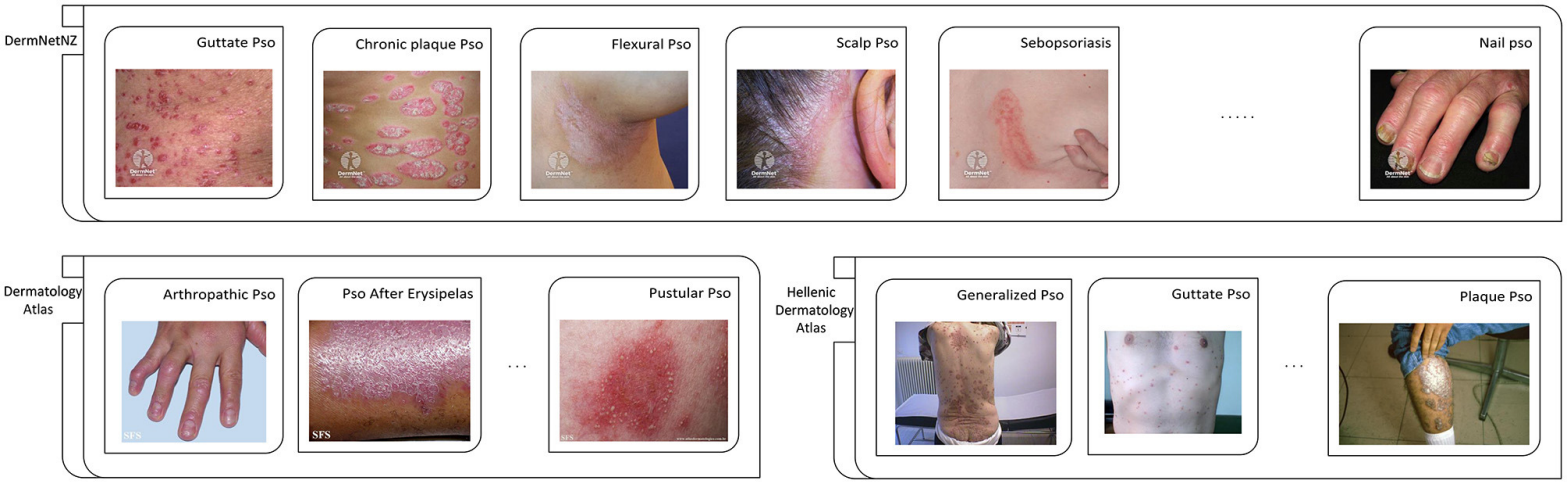
Partial examples of images from each exposed data set. Pso, Psoriasis. Reprinted with permission of six watermarked images from the DermNetNZ dataset, which is labeled as Guttate Pso, Chronic plaque Pso, Flexural Pso, Scalp Pso, Sebopsoriasis, and Nail Pso, are from https://dermnetnz.org, © DermNet^®^, licensed under CC BY-NC-ND 3.0 NZ. For the DermNetNZ dataset, the links to the individual images are as follows: Guttate Pso, https://dermnetnz.org/topics/guttate-psoriasis; Chronic plaque Pso, https://dermnetnz.org/topics/chronic-plaque-psoriasis; Flexural Pso, https://dermnetnz.org/topics/flexural-psoriasis; Scalp Pso, https://dermnetnz.org/topics/scalp-psoriasis; Sebopsoriasis, https://dermnetnz.org/topics/sebopsoriasis; Nail Pso, https://dermnetnz.org/topics/nail-psoriasis. Reprinted with permission of three watermarked images from the Dermatology Atlas dataset, which is labeled as Artropathic Pso, Pso After Erysipelas, and Pustular Pso, are from https://www.atlasdermatologico.com.br. For the Dermatology Atlas dataset, the links to the individual images are as follows: Artropathic Pso, https://www.atlasdermatologico.com.br/disease.jsf?diseaseId=43; Pso After Erysipelas, https://www.atlasdermatologico.com.br/disease.jsf?diseaseId=397; Pustular Pso, https://www.atlasdermatologico.com.br/disease.jsf?diseaseId=398. Reprinted with permission of three images from the Hellenic Dermatology Atlas dataset, which is labeled as Generalized Pso, Guttate Pso, and Palque Pso, are from http://www.hellenicdermatlas.com/en/. For the Hellenic Dermatology Atlas dataset, the links to the individual images are as follows: Generalized Pso, http://www.hellenicdermatlas.com/en/search/advancedSearch/28/528/0/; Guttate Pso, http://www.hellenicdermatlas.com/en/search/advancedSearch/28/529/0/; Palque Pso, http://www.hellenicdermatlas.com/en/search/advancedSearch/28/535/0/.

In addition to private datasets, there are also publicly accessible psoriasis datasets summarized in Table 3. One thing to note is that these publicly available datasets for psoriasis can only be applied to recognition tasks as they do not have segmentation masks and evaluation score labels. We have showcased some images from these publicly available datasets in Figure 3. Among them, the XiangyaDerm (29) and Kaggle^1^ datasets not only include psoriasis but also cover other types of skin diseases such as Melanoma, Atopic Dermatitis, Basal Cell Carcinoma (BCC), and Benign Keratosis-like Lesions (BKL). These two datasets are primarily used for multi-class skin disease recognition rather than being limited to the study of psoriasis alone. In the DermNetNZ (30), Dermatology Atlas (31), and Hellenic Dermatology Atlas (32) databases, we can observe various types of psoriasis with examples of their categories shown in the figure. The dataset available to the public contains information on different types of psoriasis, such as chronic plaque psoriasis, facial psoriasis, flexural psoriasis, and guttate psoriasis. These datasets can be used to train models to identify various types of psoriasis. Additionally, they offer a plethora of data on other skin conditions.

**Table 3:** Public dataset related to psoriasis and their description.

**Dataset**	**Description**
XiangyaDerm (29)	It contains **107,565** clinical images, covering **541** types of skin diseases. The largest amount of data in the dataset is psoriasis, **67,066** images, accounting for **62%** of the total dataset.
Skin diseases image dataset in Kaggle (see text footnote 1)	There are **10** types of skin diseases. Among them, **2,055** cases of psoriasis were included.
DermNetNZ (30)	It contains **11** different types of psoriasis, including but not limited to facial psoriasis, nail psoriasis, scalp psoriasis, etc.
Dermatology Atlas (31)	It contains **6** different types of psoriasis, including but not limited to arthropathic psoriasis, nail psoriasis, etc.
Hellenic Dermatology Atlas (32)	It contains **15** different types of psoriasis, including but not limited to generalized psoriasis, guttate psoriasis, inverse psoriasis, etc.

It can be clearly found in the Figure 3 that the most obvious problem of the psoriasis image is the lack of standardization of the data. The lesions appear in different positions, such as skin folds, hands, and joints. Some are even found in cluttered backgrounds. Therefore, it is difficult for doctors and even researchers to be confident whether the model, when recognizing these images of lesions, is extracting features from the lesion areas, or from other, distracting elements. As discussed in Yan et al. (33), there may be the same confusion concept in images of the same category, and the model is likely to refer to this confusion concept to classify this type of lesion, which we know is incorrect. We will discuss this in detail in the Challenges section.

### 3.2 ML application in psoriasis

In this section, we thoroughly describe the collected papers and summarize them in a table according to the research methodology. We also discuss the aims and results of these papers in detail. We classify the papers based on the real-world problems they address, including segmentation, recognition, and severity assessment of psoriasis.

#### 3.2.1 Lesion segmentation

The accurate segmentation of lesion areas from skin images is essential for the development of effective computer-aided diagnosis (CAD) systems for skin diseases (34). In dermatology, common skin lesions include, but are not limited to, skin cancer, acne, eczema, and psoriasis. These lesions usually have different shapes, sizes, and colors, thus requiring specific algorithms to accurately segment them (35). Commonly used lesion segmentation methods include edge-based segmentation methods, region-based segmentation methods, and DL-based segmentation methods. Among them, DL-based methods have achieved good results in many fields due to their powerful feature extraction capabilities and adaptability. We summarize and present papers that apply ML to the task of psoriasis segmentation (Table 4).

**Table 4:** Lesion segmentation.

**Methods**	**Remarks**	**References**	**Quantity of data**	**Evaluation metrics** ^*^		
				**DSC**↑	**JI**↑	**ACC**↑
Clustering	Image segmentation of lesion images using clustering algorithms from TML models	(21)	676	0.783	0.698	0.870
		(36)	780	-	0.830	0.909
CNN	The vast majority of CNN studies on psoriasis use U-Net as a segmentation model. Some papers also modify it to improve metrics	(22)	5179	0.930	0.864	0.948
		(37)	350	0.910	0.837	0.986
		(38)	500	0.948	0.901	0.992
		(39)	255	0.655	0.536	0.976
		(40)	580	0.919	-	-
Object detection backbone	Utilize object detection models as feature extraction modules in their proposed models before performing psoriasis segmentation	(41)	400	-	-	0.972
Optimization algorithm	These studies leverage CNNs where the weights and biases are optimized using optimization algorithms, for psoriasis segmentation	(42)	4200	0.960	0.905	0.970
		(43)	-	0.970	0.920	0.980

^*^DSC, Dice Similarity Index; JI, Jaccard Index; ACC: Pixel Accuracy.

For the evaluation indicators for segmentation task, the main indicators are the Dice Similarity Index (DSC) and Jaccard Index (JI). The DSC (44) metric represents the efficiency of the segmentation model by measuring the similarity between ground truth lesion (*L*_*gt*_) and predicted segmented lesion (*L*_*p*_) (45). Whereas, the JI (46) metric provides the overlapping measure between *L*_*gt*_ and *L*_*p*_ (38). Other performance metrics such as pixel accuracy (ACC), sensitivity (SE) and specificity (SP) are also available, where ACC indicates the proportion of image pixels classified correctly. In this paper, only their ACC metrics are counted. The formulas for the performance indicators are shown in Table 5.

**Table 5:** Formulas for different performance indicators for segmentation task.

**Performance metric**	**Formula** ^*^
DSC	$DSC=\frac{2\times |L_{gt}\cap L_{p}|}{\left|L_{gt}|+|L_{p}\right|}=\frac{2\times TP}{FP+FN+\left(2\times TP\right)}$
JI	$JI=\frac{\left|L_{gt}\cap L_{p}\right|}{\left|L_{gt}\cup L_{p}\right|}=\frac{TP}{TP+FN+FP}$
ACC	$ACC=\frac{TP+TN}{TP+FP+TN+FN}$

^*^TP, ture positive; FP, false positive; TN, true negative; FN: false negative.

Upon investigation, we found that the majority of papers utilizing traditional machine learning for psoriasis segmentation tasks employ clustering model algorithms (21, 36), such as K-means (8). Clustering algorithms group similar vectors in high-dimensional space and label them as the same class, excelling in both efficiency and interpretability. However, these algorithms are primarily designed for numerical datasets, necessitating modifications to the images for their application. For instance, George et al. (21) adopted a strategy of segmenting images into superpixels of varying sizes, subsequently clustering these superpixels into lesion and non-lesion regions. Ultimately, they achieved a pixel accuracy of 86.99% on 100 test images. However, with the growth of the scale and complexity of datasets, traditional methods have become inadequate. This has led to the emergence of technologies such as DL.

U-Net (13) is a very popular DL model for medical image segmentation (47). It has demonstrated superior performance in medical segmentation tasks, capable of producing accurate segmentation results even with limited training data. Therefore, researchers favor the U-Net architecture and its variants as the backbone (22, 37, 38). Raj et al. (37) proposed a model for psoriasis lesion segmentation from the raw RGB color images having complex backgrounds and challenging surroundings. Taking advantage of residual networks and migration learning, Raj et al. (38) proposed a model with a residual encoder for segmenting psoriasis lesions from digital images with uneven backgrounds, based on U-Net. Czajkowska et al. (40) used DeepLab (48) for epidermal segmentation, which is a crucial first step for detecting changes in epidermal thickness, shape, and intensity. In psoriasis diagnosis, it is also necessary to score the elevation level of lesions. However, conventional computer vision models can only process 2D images and are not well-suited for training on 3D elevation data. Therefore, this method is worth studying.

Using object detection models as a backbone for segmentation tasks is also an alternative approach compared to using conventional segmentation models (41). Their main approach is to use object detection models [e.g., Lin et al. (41) using Mask R-CNN (49)] as a backbone such as a feature extractor for the segmentation model, followed immediately by a segmentation output branch to perform the segmentation task.

Unlike proposing new CNNs, in order to guide the training of CNNs that can move toward more excellence, Mohan et al. (42) proposed a convolutional neural network (CNN) based on the Adaptive Chimpanzee Optimization Algorithm (AChOA) for automated segmentation of psoriasis skin images, which utilizes the AChOA to optimize the weights and bias values of the CNN. Similarly, Panneerselvam et al. (43) proposed Adaptive Golden Eagle Optimization (IGEO) to tune the weights and bias parameters of the CNN.

The segmentation task plays a crucial role in the application of computer technology to the medical field. It not only helps eliminate interference from non-lesion regions, but also provides a solid foundation for subsequent recognition or assessment tasks.

#### 3.2.2 Lesion recognition

The process of diagnosing skin cancer is intricate and involves visual examination and judgment by a physician, followed by microscopic examination of a biopsy. Therefore, developing more accurate algorithms for skin lesion recognition could greatly facilitate timely diagnosis of skin cancer. Automated classification of lesions is used in clinical examination to help physicians and allow rapid and affordable access to lifesaving diagnoses (50). Lesion recognition aims to differentiate psoriasis from other common skin diseases (or healthy skin) or to distinguish between different types of psoriasis, primarily through techniques such as feature extraction and segmentation. We summarize and present papers that apply ML to the task of psoriasis recognition (Table 6).

**Table 6:** Lesion recognition.

**Methods**	**Remarks**	**References**	**Quantity of data**	**Evaluation metrics** ^*^		
				**ACC**↑	**F1**↑	**AUC**↑
PCA; SVM	Traditional machine learning methods.	(23)	540	1.0	-	1.0
		(51)	90	0.90	-	-
CNNs	Classify psoriasis vs. other skin disease (including healthy skin)	(52)	1,358	-	-	0.922
		(53)	3,570	0.801	-	-
		(54)	312	0.942	0.942	0.990
		(55)	1,876	0.910	-	-
		(56)	2,101	0.919	0.894	0.959
	A publicly available dataset was used for the study.	(57)	938	0.653	0.655	0.904
		(24)	8,021	0.960	-	0.981
	Identify psoriasis from skin lesion such as eczema and pityriasis rosea that are extremely similar to it.	(58)	4,740	0.959	-	0.987
		(59)	11,031	0.920	-	-
		(60)	292	0.896	-	-
		(25)	3,732	0.962	0.958	0.971
		(61)	869	0.857	-	-
	Identify nail psoriasis from healthy nails.	(62)	1,155	0.957	-	-
	Light-weighted CNN	(63)	33,904	0.70	-	-
	CNN + ViT	(64)	8,000	0.977	0.965	-
	Classify different types of psoriasis.	(65)	30,000	-	0.890	0.920
		(66)	1,836	0.987	0.958	-
		(56)	814	0.933	0.919	-
	CNN vs. LSTM	(67)	1,838	0.842	-	-
	Light-weighted CNN	(68)	12,015	0.998	-	0.99

^*^ACC, Accuracy; F1, F1-Score; AUC, Area Under Curve.

Four performance metrics are used to evaluate the performance of the recognition models: Accuracy(ACC), recall, precision and F1-score(F1). We summarize the ACC and F1 in the paper (since F1 then already makes use of recall and precision). The formulas for the performance indicators are shown in Table 7. In addition, we also summarized the Area Under Curve(AUC) metrics from the papers. In the task, “psoriasis” was represented as a positive category and “non-psoriasis” as a negative category, and a threshold was set to distinguish positive or negative cases. By constantly adjusting this threshold, we were able to obtain multiple sets of different sensitivities and specificities. These sets were then labeled in coordinates and Receiver Operating Characteristic (ROC) curves were plotted (24). AUC is the area of the ROC curve, which is used to measure the performance of machine learning algorithms for “classification problems” (generalization ability).

**Table 7:** Formulas for different performance indicators for recognition and assessment task.

**Performance metric**	Formula^*^
ACC	$ACC=\frac{TP+TN}{TP+FP+TN+FN}$
Recall	$Recall=\frac{TP}{TP+FN}$
Precision	$Precision=\frac{TP}{TP+FP}$
F1-Score	$F1=2\times \frac{Precision\times Recall}{Precision+Recall}$

^*^TP, ture positive; FP, false positive; TN, true negative; FN, false negative.

When using TML models for psoriasis classification, researchers extract color and texture features from the images, corresponding to the erythema and silver desquamation attributes of psoriasis, respectively, since these models cannot actively analyze images (23, 51). Among them, Texture features are the most traditional way to explore specific pattern information in images, and they can quantify the texture present in lesions. Common texture analysis techniques include: Gray Level Co-occurrence Matrix(GLCM), Gray Level Run Length Matrix (GLRLM) (69), etc. For the obtained features, they can be fed into Principal Component Analysis (PCA) (70) for dimensionality reduction, which is a feature dimensionality reduction technique. From the experimental results of Shrivastava et al. (23), the best classification result was obtained by using the features of Higher Order Spectra (HOS) (71), texture and color together for classification, and the binary classification accuracy can reach 100%.

However, to achieve classification between different skin diseases, or even between different types of psoriasis, it is not enough to use TML. From the CNNs section of the table we can see that there are two main tasks in psoriasis recognition. For the former, the focus of the psoriasis identification task is on distinguishing psoriasis from skin diseases that are very similar to psoriasis compared to common classification tasks such as the ISIC dermatology dataset (72), e.g., to distinguish scalp psoriasis from scalp seborrheic, which have the same region of onset and a small difference in the lesion appearance but have completely different treatment approaches, CAD comes in handy in order to avoid incorrect diagnoses by doctors (52). Lichen planus, parapsoriasis, lupus erythematosus and eczema are also particularly similar but differently treated skin conditions which, in addition to all being characterized by a reddish color, also have papules or plaques (25, 58–61). Because of Inflammatory skin diseases, such as psoriasis (Pso), eczema (Ecz), and atopic dermatitis (AD), are very easily to be mis-diagnosed in practice, Wu et al. (58) developed an end-to-end deep learning model. Yang et al. (59) aimed to train an efficient deep-learning network to recognize dermoscopic images of psoriasis (and other papulosquamous diseases), improving the accuracy of the diagnosis of psoriasis. While they have similar symptoms, Psoriasis and Eczema have vastly different underlying causes and behaviors, Chatterjee et al. (60) explores state of the art Deep Learning techniques for distinguishing Psoriasis and Eczema. Hammad et al. (25) presents an enhanced deep learning approach for the accurate detection of eczema and psoriasis skin conditions. Zhu et al. (61) propose a novel abscissa-ordinate focused network (AOFNet) with active label smoothing for the identification of psoriasis and eczema from images.

Using models from the natural language processing (NLP) domain to extract image features is a very popular approach. This is because these models, when applied to sentences, are able to capture the distant relationships between sentences and thus calculate the relationships between words. The researchers want to try to use this idea to capture long distance relationships between images to make up for the fact that the computation of convolution can only capture local information. Aijaz et al. (67) innovatively used Long Short-Term Memory (LSTM) (73) for classification in addition to CNNs. However, LSTM only obtained an accuracy of 0.723 on the results (CNN obtained 0.842), proving that CNN is still superior to models from NLP for image processing. Vishwakarma et al. (64) proposed a model that combines the features of a CNN and a Vision Transformer (ViT) (74) with the aim of building a high-performance, lightweight hybrid model for the intended task. In this, ViT processes the convolutional feature maps to capture long-term dependencies that represent global features.

The use of deeper neural networks is a straightforward and effective way to deal with the increase in the amount of data, but this can lead to a very fatal problem - an increase in the number of parameters, resulting in the need for better hardware. However, instead of opting for a larger model, Arunkumar et al. (63) proposed their own lightweight CNN when solving tens of thousands of datasets, and obtained relatively good results. The model proposed by Rashid et al. (68) is very easy to be used and deployed as a smartphone application in a real-time decision-making environment due to its lightweight nature. The model can handle recognition and classification of psoriasis types for low or high resolution images.

Zhao, Aggarwal, and Rashid et al. (24, 57, 68) used the psoriasis dataset (Table 3) from a public dataset for identification of common skin diseases and psoriasis. The study using the public dataset can enhance the confidence of the diagnosis as all images were verified by pathological examination and history and labeling was done by experienced dermatologists. We believe that psoriasis research will become more comprehensive as more and more papers conduct research on public datasets.

#### 3.2.3 Lesion severity assessment

Psoriasis severity assessment refers to the objective and accurate evaluation of the severity of a patient's psoriasis, so that the doctor can develop a reasonable treatment plan and monitor its effectiveness. Commonly assessment methods include the PASI scoring system, DLQI scoring system (75), etc. Among them, the PASI score system is used to score psoriasis patients based on factors such as lesion area, erythema, scaling, and infiltration, with a total score of 0 to 72. The higher the score, the more severe the condition. In the process of using ML to evaluate the severity of psoriasis, feature selection is a very important step, including the extraction of features such as lesion area, erythema, scaling, and infiltration. Before this, it is necessary to segment and identify the image, especially to prevent the background interference from affecting the extraction of color features. We summarize and present papers that apply ML to the task of psoriasis severity assessment (Table 8).

**Table 8:** Lesion severity assessment.

**Methods**	**Remarks**	**References**	**Quantity of data**	**Evaluation metrics** ^*^		
				**ACC**↑	**F1**↑	**AUC**↑
PCA; SVM; NB; DT	Traditional machine learning methods	(76)	17	0.920	-	-
		(26)	848	0.999	-	0.999
		(27)	670	0.998	-	0.998
Dic. L; BoVWs	A novel image representation and unsupervised feature extractor method	(77)	676	-	0.710	-
		(78)	676	0.808	-	-
CNNs	Segmentation was performed before severity assessment	(28)	5,000	0.926	0.926	0.992
	Semi-automatic vs. automatic segmentation algorithms	(79)	80	-	0.989	-
	Segmenting and scoring nail psoriasis	(80)	705	0.765	-	-
		(81)	300+	0.915	-	-
		(82)	1,154	0.55	0.55	0.63
	Segmenting and scoring pustular psoriasis (PP)	(83)	611	0.667	-	-
	Segmenting and scoring large areas of psoriasis	(84)	500	0.942	-	-
	CNN + ViT	(85)	1,018	0.795	0.792	0.950
	Direct assessment of psoriasis severity using CNNs	(86)	705	-	-	-
		(87)	1,731	-	-	-
		(88)	14,096	-	-	-
		(89)	5,951	-	0.940	-
		(90)	792	0.910	-	-
		(91)	2,700	-	-	-
	Attention	(92)	792	0.908	0.930	-
	YOLO	(93)	2,657	-	-	-

Similar to the psoriasis classification task, the task of psoriasis severity assessment using TML models also requires the extraction of various features such as color and texture in the image, which are then fed into various classifiers for severity assessment. In this regard, Shrivastava et al. (26, 27). conducted two different experiments on two different datasets, one on the 848 psoriasis dataset, which achieved 99.92% accuracy, and one on the 670 psoriasis dataset, which was first segmented by Bayesian modeling and then classified, which achieved 99.84% accuracy. It can be noticed that although the dataset has become smaller, the accuracy can still be kept high by segmentation followed by classification.

In the experiments of Moon et al. (79), they used and compared automatic [Simple linear iterative clustering (SLIC) superpixel-based segmentation (21) and U-Net model] and semi-automatic [level set method (LSM) (94) and interactive graph cuts (IGC) (95)] segmentation algorithms. It was found that the semi-automatic segmentation models are particularly subjective and time consuming, while the automatic models are less effective in segmenting the curved, illuminated or shadowed parts of the image. From the results, the LSM from semi-automated segmentation was able to achieve a DICE of 0.945 and the SLIC from automated segmentation a DICE of 0.915 (Other segmentation metrics are noted in the paper). Taking into consideration time efciency and reproducibility, the paper finally chose SLIC as the segmentation task model before the evaluation task.

The work of Dash et al. (28) is the most consistent with the physician's diagnostic process within all the papers. Specifically, they distinguished 5,000 healthy skin from 5,000 psoriasis with 99.08% accuracy, then, segmented the lesion areas in the psoriasis images with 94.76% accuracy, and, ultimately, assessed the segmented images at four levels of severity with 99.21% accuracy. Raj et al. (84) extended the work of Dash et al. (22) by broadening the scope of lesion detection to segment healthy skin, psoriatic lesions, and background regions simultaneously from full-body areas.

Training out a segmentation model requires relevant data with labels, and how well it is trained affects the subsequent tasks, with errors at each stage accumulating to be very catastrophic in the end (77). Thus, Huang et al. (88) avoided the use of segmentation models and instead added various attention modules after the backbone output, allowing the model to localize the lesion area without going through the segmentation model. Schaap et al. (87) utilized a special CNN (96) for the assessment task. This CNN is assessed for psoriasis with a decreasing probability from 0 to 5, with a final threshold set to arrive at a score for that psoriasis. Moon et al. (92) used CutMix to generate multiple-severity disease images and proposed a hierarchical Multiscale Deformable Attention Module (MS-DAM) that adaptively detects representative regions of irregular and complex patterns in multi-severe disease analyses.

You Only Look Once (YOLO) (97) is a deep neural network-based target recognition and localization algorithm with fast processing speed and suitable for real-time systems. YOLO-v4, which builds on the original YOLO target detection architecture, employs state-of-the-art optimization strategies in the field of CNNs. Thus, Yin et al. (93) used the YOLO-v4 algorithm as a feature extractor for images to detect the severity and lesion area of each disease in a specific portion of an image and perform a comprehensive assessment.

ViT's input adaptive weighting and global information learning can show good performance in vision related tasks. Raj et al. (85) put ViT into a classification module for computation, where the feature vectors output from the backbone are computed globally, and then the output is collapsed back into the dimensions of the feature representations produced by the convolution operation.

## 4 Discussion

### 4.1 Methods statistical analysis

**Figure 4:**
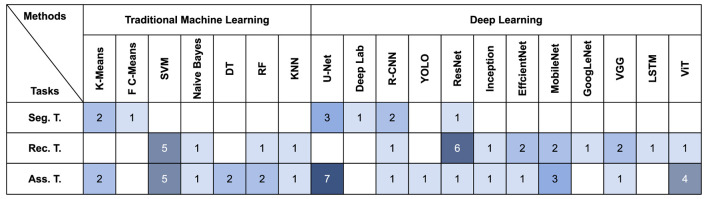
Quantitative distribution of different ML methods on the three tasks.

We have summarized the methods used in the collected papers (Figure 4). We found that when researchers select TML models, for segmentation tasks, clustering models such as K-Means are usually used to achieve segmentation of diseased regions by clustering diseased pixels together. Whereas for lesion recognition and assessment tasks, given the limited datasets available for psoriasis, researchers tend to favor support vector machines as it performs well with small datasets.

In DL model selection, U-Net is widely used for its high accuracy in medical segmentation (98). Segmentation models are also utilized in psoriasis recognition or assessment tasks, where only by locating and segmenting the diseased regions, the model is able to avoid interference from non-diseased regions (99).

Some methods originally used for NLP (e.g., LSTM and Transformer) have been widely used in the field of computer vision in recent years (100), and have also been applied to medical image analysis. However, there are fewer papers using these methods to analyse psoriasis, and their scalability in medical images needs to be further investigated. In addition, many other methods are not shown in the diagram, and we have only summarized the most commonly used ones.

### 4.2 Challenges

Through a comprehensive analysis of collected papers, including data collection, preprocessing, modeling approaches and experiments, we analyse the current challenges of machine learning in psoriasis.

#### 4.2.1 Lack of data sources

ML (especially DL) algorithms require large amounts of data to effectively train models (101). However, since very few people study psoriasis in the field of ML, the amount of data available for analysis then becomes very limited, making it difficult to build accurate and reliable models. In addition, most psoriasis datasets are not publicly available, and most of the datasets used in the papers listed in the table above were obtained through collaboration with hospitals. Moreover, different tasks require different annotations, which adds to the complexity of ML for research in the field of psoriasis. To use ML for psoriasis research, access to sufficient data is critical. However, this may not always be feasible due to the high cost of physician annotation time or the difficulty of obtaining consistent images (102). In addition, the acquired images may have unevenly distributed categories or incorrect labels, which can lead to training the model in the wrong direction or overfitting.

#### 4.2.2 Data inconsistency

Even if there is enough data, its inconsistency and irregularity can lead to poor model performance. That is, if the data come from different databases or are taken by different doctors with different angles, lighting or resolutions, then the integration and analysis of these data will be a big challenge. Although the International Skin Imaging Collaboration (ISIC) has attempted to address the issue of data standardization by developing a set of technical standards for skin lesion imaging (103), psoriasis differs from common dermatological datasets in that the site of onset can be systemic (e.g., body depressions), which leads to the analysis not being able to train the model exactly according to the characteristics of the dermatological condition (rounded, localized, more regular, flattened). At the same time, some features are difficult to obtain through machine such as the sclerotic height of psoriasis, and most of the commonly used DL is applied to flat images, which can only obtain features that are accessible to flat vision, such as color and texture. Although skin thickness segmentation was proposed in Czajkowska et al. (40), it is particularly demanding on the dataset.

#### 4.2.3 The inexplicability of methods

Selection of appropriate methods and improvement of existing methods to improve the accuracy of psoriasis analyses are common threads in existing papers, but doctors and patients are most concerned about the accuracy of psoriasis analyses and whether the researchers can explain how the proposed models arrive at their conclusions. However, from the collected papers, most of them only propose a model with good diagnostic results for psoriasis, while little research has been done on the interpretability of the model.

### 4.3 Future development

In response to these challenges to the application of ML in psoriasis, we propose solutions and summarize the future development of ML.

#### 4.3.1 Few-shot learning

Model training using a small amount of data is also a current research hotspot in ML, especially DL. For example, Folle et al. (82) used a small number of samples to study the diagnosis of psoriasis, and the BEiT model, which they used, was designed to train models with fewer samples. Few-shot learning is a ML paradigm designed to enable efficient training of models with a small number of samples. In Xiao, Liu and Chen et al. (104–107), they classified and segmented lesion data with fewer lesion images. Data collection for psoriasis is also difficult, especially labeling, and requires overcoming a variety of subjective factors. In today's era of predominantly data-driven model training, smaller, more granular datasets may produce better results than larger, more extensive datasets.

#### 4.3.2 Feature consistency

Differences between images can also worsen the model, especially in feature extraction. Therefore, we would like to unify the images before training the model, or, in other words, extract common features. For example, Diaz et al. (108) aim to pixelate images using a segmentation model that labels pixels belonging to the same lesion features (e.g., pigment networks, blue-white stripes, dots, bubbles, blood vessels) as belonging to the same category in skin lesions. This reduces the differences in image-level features by extracting pixel-level features, while directing the model to use these features for further training and avoiding image differences that cause the model to recognize the same features as different features. However, segmentation requires labeling, which leads to a relatively poor feasibility of this approach. To solve this problem, Pathak et al. (109) used the idea of weak segmentation, which does not require prior labeling, but automatically obtains the segmentation labels through learning. Using this idea, when faced with psoriasis images that are extremely different at the image level, the model can recognize the same attributes or features between them, thus enabling the model to better assess psoriasis. In addition, preprocessing features of skin lesions (e.g. color) is also an aspect that could be considered. Barata et al. (110–112) have shown that image preprocessing techniques (e.g. color constancy) can improve the performance of AI systems for segmentation and classification of skin lesions. Using such techniques, when assessing the severity of a feature of psoriasis (e.g. erythema), it may be possible to avoid situations where the assessment of erythema is different due to the difference in the psoriasis, if we can first normalize the psoriasis.

#### 4.3.3 Model explainability

Currently, there is an increasing amount of interpretable research in the field of AI in medicine (113). These papers essentially use techniques that are intuitively capable of interpreting the model to enable interpretable research. For example, a class activation map (CAM) (114) is used to visualize the regions of interest of the model, just as Ding et al. (115) used a CAM to direct the model's attention to the lesion region while explaining the model's focus in the middle layer. Concept activation vectors (CAV) (116), a technique that converts high-level concepts that can be understood by humans (e.g., whether or not there are hairs in the area of the lesion, etc.) into vectors that can be understood by a computer. It is therefore feasible to use CAM or CAV to interpret the model. Using CAM, it is possible to understand which areas on the image the model focuses on, and using CAV, it is possible to direct the model's attention to which important high-level concepts. Of course, there are many more interpretable techniques waiting to be discovered, all aimed at increasing physician or patient trust in the model and its outputs.

## 5 Conclusion

This review provides an overview of the application of ML (especially DL) to psoriasis diagnosis over the last decade, including segmentation, recognition and assessment tasks. However, we have identified a number of challenges in this area, the most important of which are data inconsistency and the issue of data privacy. It is also worth noting that not all DL models are best suited for every task. TML algorithms have also shown good results in feature extraction, and different models should be selected depending on the specific task at hand.

In conclusion, we hope that this review will encourage research in this area and stimulate more advanced techniques to help physicians in their work.

## Data availability statement

The original contributions presented in the study are included in the article/supplementary material, further inquiries can be directed to the corresponding author/s.

## Author contributions

HL: Funding acquisition, Supervision, Writing – review & editing. GC: Methodology, Visualization, Writing – original draft. LZ: Conceptualization, Data curation, Methodology, Writing – original draft. CX: Funding acquisition, Supervision, Writing – review & editing. JW: Resources, Writing – review & editing.

## Footnotes

^1^ https://www.kaggle.com/datasets/ismailpromus/skin-diseases-image-dataset
